# Placental Implications of Peroxisome Proliferator-Activated Receptors in Gestation and Parturition

**DOI:** 10.1155/2008/758562

**Published:** 2008-01-20

**Authors:** Valerie Borel, Denis Gallot, Geoffroy Marceau, Vincent Sapin, Loïc Blanchon

**Affiliations:** ^1^Université d'Auvergne, JE 2447, ARDEMO, 63000 Clermont-Ferrand, France; ^2^INSERM, U.384, Laboratoire de Biochimie, Faculté de Médecine, 63000 Clermont-Ferrand, France; ^3^CHU Clermont-Ferrand, Maternité, Hôtel-Dieu, 63000 Clermont-Ferrand, France

## Abstract

The placenta is a transitory structure indispensable for the proper development of the embryo and fetus during mammalian gestation. Like other members of the nuclear receptor family, the peroxisome proliferator-activated receptors (PPARs) are known to be involved in the physiological and pathological events occurring during the placentation. This placental involvement has been recently reviewed focusing on the early stages of placental development (implantation and invasion, etc.), mouse PPARs knockout phenotypes, and cytotrophoblast physiology. In this review, we describe the placental involvement of PPARs (e.g., fat transport and metabolism, etc.) during the late stages of gestation and in the amniotic membranes, highlighting their roles in the inflammation process (e.g., chorioamnionitis), metabolic disorders (e.g., diabetes), and parturition.

## 1. THE PEROXISOME PROLIFERATOR-ACTIVATED RECEPTORS (PPARs)

### 1.1. Nomenclature and structure

**Figure 1:**
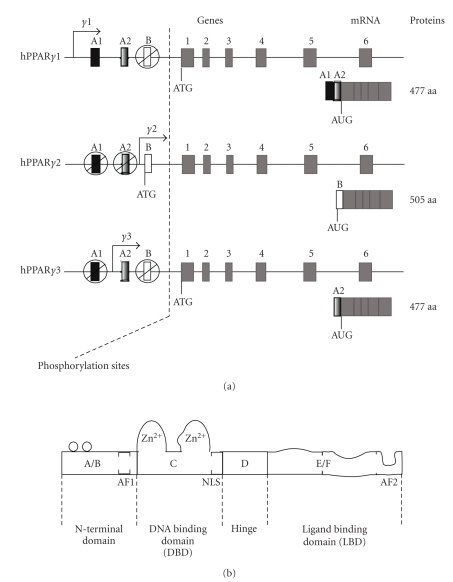
(a) Schematic representation of PPAR*γ* genes, mRNA, and proteins. The $5^{\prime }$exons A1, A2, B can be alternatively spliced to give rise to the different PPAR*γ* isoforms. The boxes 1 to 6 correspond to exons which are common to PPAR*γ*1, *γ*2, *γ*3 genes. ATG is the initiation transcription site. The molecular weight of these isoforms ranges from 49 to 56 kDa. (b) Schematic representation of typical nuclear receptor structure. AF1: activating function 1 (ligand-independent function), AF2: activating function 2 (ligand-dependent function), NLS: nuclear signal localization.

Discovered in 1990, PPARs are known for their biological role in inducing the proliferation of peroxisomes in rodents [1]. They are transcription factors belonging to the ligand-activated nuclear hormone receptor superfamily [2] and have been identified in different species such as the xenopus, mouse, rat, and humans. In all these species, PPARs present three isotypes encoded by distinct single-copy genes: PPAR*α* (NR1C1), PPAR*β*/*δ* (also called NUC1 or NR1C2), and PPAR*γ* (NR1C3), located on chromosomes 15, 17, 6 in the mouse and chromosomes 22, 6, 3 in humans, respectively. The PPAR*γ* gene alternative promoters give rise to three different isoforms named *γ*1, *γ*2, and *γ*3 which differ at their 5′ends (see Figure 1(a)) [3]. PPAR *α*, *β*, *γ*1/*γ*3, *γ*2 translation produces proteins of 468, 441, 475, and 505 amino acids, respectively, with a molecular weight of 49 to 56 kDa [4]. By performing multiple PPAR nucleotide/protein alignments of PPARs in different species, a strong interspecies identity (human, mouse, rat, bovine, ≈90%) has been established, illustrating a strong evolutionary conservation among species by derivation from a common ancestor (Table 1). PPAR*γ* shows the highest conservation in terms of cDNA and proteins.

Like several other members of the nuclear receptor superfamily, PPARs possess the typical structure organised in six domains named A to F (see Figure 1(b)) [5]. Domain C (DBD: DNA binding domain) contains two zinc fingers and allows promoter target gene interaction and dimerization with its preferential nuclear receptor: retinoid X receptor (RXR). The PPAR/RXR heterodimer binds to the target gene promoter response element named peroxisome proliferator response element (PPRE) which is made up of two half site AGGTCA separated by one or two nucleotides (also called DR1 or DR2 for direct repeat 1 or 2) and a $5^{\prime }$ extension A (A/T) CT. Domain E/F allows ligand binding and contains a ligand-dependent transactivation function called AF2 (activating function 2). It is involved in dimerization and interaction with cofactors.

### 1.2. PPAR ligands

As with the other nuclear receptors, the binding of the ligand is a key step in the control of PPAR transcriptional activity. In the absence of a ligand, corepressors and histone deacetylases (HDAC) bind to PPARs and inhibit the transcription activation of target genes. PPAR ligands have the ability to dissociate the corepressor complexes from the PPAR/RXR heterodimer, allowing the binding of the coactivators in order to initiate and activate transcription.

There are two kinds of ligands for the PPARs: natural and synthetic. Among the natural ligands the monounsaturated fatty acids (FA) (e.g., oleic acid) and the polyunsaturated fatty acids (PUFA) (e.g., linoleic acid, linolenic acid, and arachidonic acid) are described as ligands for PPAR*α*, PPAR*β*, and PPAR*γ*. They act with concentrations consistent with those found in human serum [6]. The different PUFA metabolites: 8(S)- and 15-hydroxyeicosatetraenoic acid (8(S)- and 15-HETE), leukotriene B4 (LTB4), 9- and 13-hydroxyoctadedienoic acid (9-HODE and 13-HODE) and 15-deoxy-Δ^12, 14^-prostaglandin J2 (PGJ2) are potent selective activators of PPAR*α* and PPAR*γ*. Some oxidized low-density lipoproteins (LDLs), oxidized alkyphospholipids, nitrolinolenic acid, and prostaglandin metabolites can also activate PPAR*γ* [7]. Recently, it has been demonstrated that P450 eicosanoids are potent PPAR*α* and PPAR*γ* ligands [8]. Indeed, Ng et al. [8] have shown that P450 catalysed arachidonic acid metabolites like 20-hydroxyeicosatetraenoic acid (20-HETE) or 11, 12-epoxyeicosatrienoic acid (11, 12-EET) can activate PPAR*α* and PPAR*γ*. These ligands induce PPAR binding to PPRE and can modify the expression of PPAR*α* responsive genes like apoA-I or apoA-II in the same way than synthetic ligands. Thus the finely regulated conversion of PUFAs to eicosanoids through either the lipoxygenase, cyclooxygenase, or cytochrome P450 monooxygenase pathways may provide a mechanism for the differential regulation of PPAR*α* and PPAR*γ* and their respective target genes. PPAR*β* can be activated by different types of eicosanoids including prostaglandinA1 (PGA1) and prostaglandin D2 (PGD2). Many synthetic ligands exist and have been used in PPAR work. These ligands include prostaglandin 12 analogs, pirinixic acid (Wy-14643) for PPAR*α*, hypolipidemic and hypoglycemic agents (nonthiazolidinedione) for PPAR*β*, and thiazolidinediones (e.g., rosiglitasone, troglitazone) for PPAR*γ* [2].

## 2. PPAR EXPRESSION PATTERNS

**Figure 2:**
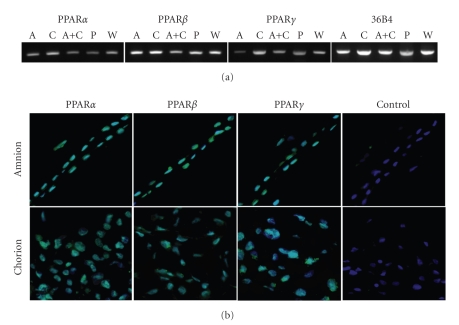
PPARs expression in term placenta and amniotic membrances. (a) RT-PCR assays of PPAR*α*, PPAR*β*, and PPAR*γ* mRNA in amnion, chorion, placenta, and WISH cells. PCR products were analyzed on 1.8% agarose gel and stained with ethidium bromide. 36B4 corresponds to the housekeeping gene. A: Amnion, C: Chorion, A+C: Amnino+Chorion, P: Total placenta, W: WISH cells. (b) PPARs immunostaining of amnion and chorion. Note that all PPARs are expressed in nucleus. Magnification: x200.

The adult PPAR expression patterns have been extensively established at the mRNA and protein levels in several species (Table 2) [8, 9]. Several studies conducted during mammalian gestation have established the placenta as an important expression site of the different PPARs isoforms. Our review will focus only on term placental expression and on the amniotic/fetal membranes. The placental dynamic expression of the 3 PPARs during early and midgestation (of mouse, rat, and human) is well described in Fournier et al., 2007 [4]. In rat placenta, all three PPAR isoforms are ubiquitously expressed from 11 days postcoitum (dpc) [10]. Both PPAR*β*/*δ* and PPAR*γ* are expressed after 8.5 dpc in mouse placenta. By immunohistochemistry and RT-PCR, the three PPAR isoforms are been shown to be expressed in the villous trophoblastic cells and syncytiotrophoblasts of the human term placenta [4]. To extend the previously published results [11] and to assess the potential importance of PPAR proteins in fetal membranes, RT-PCR and immunohistochemistry experiments were performed on human term placental samples. The three PPARs are present in total placenta, amnion, chorion, and in amnion-derived WISH epithelial cell line at the mRNA (see Figure 2(a)) and protein levels (see Figure 2(b)). The expression of PPAR*α* and PPAR*γ* seems to be weaker than that observed for PPAR*β*/*δ*. In addition, a greater amplification of the PPAR*γ* cDNA is obtained in chorion than in amnion, where PPAR*γ* is almost undetectable.

## 3. IMPLICATIONS OF PPARs IN PLACENTA AND FETAL MEMBRANES

### 3.1. Placental and amniotic presence of PPARs ligands

The lipids of human amnion and chorion are enriched in the essential fatty acid arachidonic acid, which is the precursor of all the prostaglandins of the 2 series [13]. Sixty-six percent of the arachidonic acid of the human fetal membranes are available in the glycerophospholipids of these tissues and can easily be converted into PGD_2_ [14]. The placenta produces considerable amounts of PGD2 [15]. The enzymes necessary to convert PGD_2_ into prostaglandin J2 (PGJ2) are present and coexpressed with PPAR*γ* in placenta. 15-Deoxy-Δ^12, 14^-PGJ2 (15dPGJ2) and its precursor PGD2 are present in amniotic fluid at concentrations that do not exceed 3 nM [16]. However, this amniotic fluid concentration cannot be an exact representation of the physiological placental reality for PPARs ligands because the nuclear concentration is not measured. The maternal blood may also be a source of PPAR ligands for the human placenta and the fetal membranes. It has been established that a heat-stable compound (not a protein, but rather a prostanoid or a fatty acid) is detected in maternal blood serum and is able to activate the PPAR*γ* [17]. The presence of classical and new PPARs ligands (e.g., P450 eicosanoids, PUFA metabolites) in placenta and fetal membranes suggests that they could activate PPAR, induce PPAR binding to PPRE, and modify the expression of PPAR target genes; but this hypothesis has to be confirmed by further analysis, based on PPARs activation in other organs. For example, PUFAs, such as and eicosapentaenoic acid (EPA) and docosahexaenoic acid (DHA), increased PPAR*γ* mRNA expression and binding to PPRE in renal tubular epithelial cell line (HK-2). Furthermore, they downregulate LPS-induced activation of NF-*κ*B via a PPAR*γ*-dependent pathway in HK-2 cells [18]. Another example showed that PGD2 is among the most abundantly produced prostaglandins in synovial fluid by synovial fibroblasts [19]. It can be converted into PGJ2. It has been demonstrated that PPAR*γ* ligands (15dPGJ2) inhibit IL-1*β*–induced production of nitric oxide (NO) and matrix metalloproteinase-13 (MMP-13) in chondrocytes. This inhibition was PPAR*γ*-dependent and occurred at the transcriptional level, through repression of NF-*κ*B signalling [20]. These two examples support a role of PPAR ligands in fetal membranes.

### 3.2. Fundamental implications of PPARs during early placentation

As a determining result, the knockout of the PPAR*γ* in mice [21] yielded the first findings indicating the importance of this factor in early embryonic and perinatal development. These results are concomitant with those obtained by the generation of RXR*α* or *β* null mice (PPAR*γ* partner in the functional heterodimer), also showing an embryonic lethality explained by the lack of generation of a functional labyrinthine zone [22]. Furthermore, complementary studies conducted by the inactivation of PPAR*γ* coactivators or coregulators, such as peroxisome proliferators activator receptor-binding protein (PBP) and peroxisome proliferator-activated receptor-interacting protein (PRIP), also lead to severe placental dysfunction, such as inadequate vascularisation of the structure [23–25]. Recently, Barak et al. also demonstrated that the inactivation of PPAR*β*/*δ* led to the formation of abnormal gaps and a thinner but fully differentiated vascular structure in the placentodecidual interface [26]. These results establish the nonredundant roles of PPAR*γ* and PPAR*β*/*δ* in early mouse placental development. By contrast, the inactivation of PPAR*α* has no effect on placental formation or on the developing foetus and by the way theirs possible roles during pregnancy had to be clarified [2]. In humans, the studies are almost exclusively focused on the PPAR*γ* roles during early placentation. It has been clearly established that all three PPARs can stimulate or inhibit the differentiation and/or proliferation of the villous cytotrophoblasts into syncytiotrophoblasts and the synthesis of chorionic gonadotrophic hormone, and may hamper extravillous trophoblastic cell invasion (for more details, see Fournier et al., 2007 [4]).

### 3.3. Roles of PPARs in the uptake and transport of trophoblastic lipids

As one of the first functions described for PPAR*γ* in other tissues, trophoblastic lipid uptake and accumulation are also regulated in part by this factor [27]. The PPAR*γ* ligands seem to increase the uptake and accumulation of the fatty acids in human placenta [28]. This regulation is associated with an enhanced expression of adipophilin (fat droplet-associated protein) and fatty acid transport proteins (1 and 4) in human trophoblasts [28–30]. These results were confirmed recently by the in vivo activation of PPAR*γ* by its agonist rosiglitazone in mice, which also leads to the enhancement of the previous described genes plus two new ones involved in the lipid transport: S3-12 (plasma associated protein) and myocardial lipid droplet protein/MLDP [27]. Taken together, these results confirm the results obtained on PPAR*γ*-null mutants: the absence of the lipid droplets normally present around the fetal vessels in the wild-type placenta [21].

### 3.4. PPARs in placental inflammatory response and in the parturition signalling

**Figure 3:**
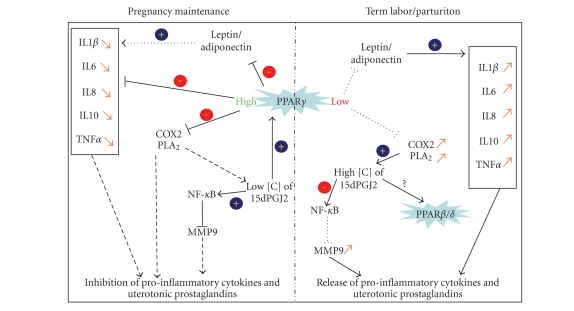
Schematic representation of PPAR*γ* implication in pregnancy maintenance and labor. IL1*β*: Interleukin 1*β*; IL6: Interleukin 6; IL8: Interleukin 8; IL10: Interleukin 10, TNF*α*: Tumor Necrosis Factor *α*; COX2: Cyclo-oxygenase type 2; PLA_2_: Phospholipase A2; NF-*κ*B: Nuclear Factor-Kappa B; MMP9: Matrix Metalloproteinase 9; 15dPGJ2: 15-Deoxy-Δ12, 14-prostaglandin J2.

At this stage of our knowledge of PPARs, the most interesting results have been obtained with the study of their involvement in the inflammation process, which may be linked to labor at term and also to the premature rupture of fetal membranes (see Figure 3). Term labor is associated with an increase in proinflammatory proteins and cytokines such as IL1*β*, IL6, IL8, IL10, and TNF-*α*. This increase in proinflammatory proteins and cytokines induces uterine contractions. PPAR*γ* ligands have been demonstrated to inhibit the secretion of IL6, IL8, and TNF-*α* in amnion and chorion [31], highlighting the role of PPARs in the regulation of the inflammatory response in human gestational tissues and cells [32–35]. The parathyroid hormone-related protein (presenting a cytokine-like action) is involved in many processes during normal and pathological pregnancies, and is decreased by PPAR*γ* stimulation [36], which also blocks proinflammatory cytokine release by adiponectin and leptin [37]. The production of prostaglandins by the endometrium, the myometrium, and the fetal membranes induces the contraction of the myometrium during labor. This generation of uterotonic prostaglandins correlates with the increased prostaglandin-endoperoxide synthase type 2/cyclooxygenase type 2 (COX-2) activity and the increased secretory phospholipase A2-IIA (sPLA2) mRNA, proteins and activities. By inhibiting the production of the COX-2 and sPLA2 in fetal membranes, PPAR*γ* promotes the quiescence of the uterus during gestation [34]. The molecular action of 15dPGJ2 seems to involve interactions of the NF-Kappa B signaling pathway, inducing reduction of PGF2*α*, PGE2, and MMP9 release in the placental environment [31]. This suppressive action of PPAR*γ* on inflammation is apparently time-dependent during pregnancy. The PPAR*γ* level of expression remains stable throughout gestation, except for the period just before labor, when its expression in fetal membranes declines. This reduction is coincidental with a relative increase in COX-2 expression [38]. Further work has shown this simple scheme to be more complex. While the expression of PPAR*α* does not change at term in amnion, it decreases in chorion. An increase was also demonstrated for PPAR*β*/*δ* in chorionic and amniotic zones [11]. These last two findings raise the question of the involvement of the *α* and *β* isoforms in this process. The absence of a real link between COX-2 and PPAR*γ* is presented by Lindstrom and Bennett [39]. Finally, the PPAR action seems to be concentration-dependent. A small amount of 15dPGJ2 (*<*0.1 μM) acts through the PPAR*γ* signaling pathway, where at high concentration (1 μM) its actions are most probably mediated through other pathways: PPAR*β*/*δ* and/or an inhibition of NF-*κ*B independent of PPARs [35]. Furthermore, 15dPGJ2 and troglitazone were also demonstrated to have some antiinflammatory or apoptosis-induction specific effects by PPAR*γ*-independent pathways. This was suggested by the work of Lappas et al. on human gestational tissues, demonstrating that this effect could passed by antagonist effect of 15dPGJ2 on the NF-*κ*B pathways and antioxidant effects of the troglitazone, a synthetic ligand of PPAR*γ* [31].

### 3.5. PPARs in placental and amniotic membranes pathologies

In contrast to the different roles described for PPARs during human placentation, only a few studies on PPARs and placental pathologies have been conducted. In choriocarcinoma and hydatiform moles, a downregulation of the PPAR*γ* expression is observed but this real influence needs to be elucidated [40]. The potential involvement of PPAR*γ* on preeclampsia is suggested by the fact that this pathology is associated with an increased peroxidation in trophoblasts [41, 42]. An overproduction of 15-HETE has also been noted, suggesting a deregulation of PPAR*γ* [43]. This can cause a strong transactivation of PPAR*γ* during early pregnancy, resulting in a reduction of extravillous trophoblastic invasion, one cellular explanation often cited in the physiopathology of preeclampsia [44, 45]. Other abnormal transactivation of PPARs may be hypothesized to explain placental pathologies. The 15dPGJ2 has been shown to induce apoptosis of the placental (JEG-3) and amniotic (WISH) established cell line, [46, 47]. An excess of 15dPGJ2 production can be a source of placental dysfunction linked to an increase in trophoblastic death. It is also established that deletion of PPAR*γ*, PPAR*β*/*δ*, and some of their coactivators (PBP, PRIP, and RAP250) induce abnormal placental phenotypes (abruption, reduction of fetomaternal exchanges, and alterations of trophoblastic differentiation) in null mutants [21, 23, 24, 26, 48, 49]. Chromosomal and/or genetic alterations (point mutation or deletion) may occur for these genes, inducing human placental alterations. The placental 11β** hydroxysteroid dehydrogenase type 2 is a target gene of PPARs [50]. This enzyme plays a key role in fetal development by controlling fetal exposure to maternal glucocorticoids. An abnormal regulation by PPARs may result in an absence of fetal protection. In the rat placental HRP-1 established cell line, the phthalate and derivatives transactivate PPARs (*α* and *γ*) induced an increase in uptake rates of fetal essential fatty acid and the transport of arachidonic and docosahexaenoic acid [51]. If such a mechanism can be induced by the phthalates during human placentation, this may strongly affect the fetal essential fatty acid content during growth.

Gestational diabetes is linked to impaired lipids metabolism [52]. Decreased 15dPGJ2 in blood of diabetic mothers is also linked to a decrease in placental PPAR*γ* expression. The inhibition of PPAR*γ* results in an induction of a placental proinflammatory environment associated with an increase in nitrogen monoxide production and release, which can impair fetoplacental development [53, 54].

The PPAR regulation of inflammation may be very important in another obstetrical pathology of the amniotic membranes: the chorioamnionitis. This pathology, usually due to an ascendant colonization of pathogenic microorganisms from the vagina to the uterus, is closely associated with preterm labor and premature rupture of membranes (chorion and amnion). These ruptures of membranes seem to arise from deregulated proinflammatory factor synthesis. It has already been reported in this pathology that IL1*β*, IL6, IL8, TNF-*α*, and prostaglandinE(2) show inadequate concentrations in placental membrane and in amniotic fluid [55–58]. As PPARs may be involved in the occurrence and control of this inflammatory response, further studies are needed to assess their importance in this process and to find new possible therapeutic strategies to prevent this damaging pathology.

More generally, the use of natural and synthetic PPAR ligands looks to be a promising way in preventing placental pathologies such as endometriosis or preeclampsia. An interesting study also demonstrates that the reduction of LPS induction of cytokines is reduced by PPAR*γ* ligands in fetal membranes. Nevertheless, the few studies already conducted were done practically only on animal (rodent) models and looks to have positive effects on the pathologies (for review see Toth et al. [59]). Till now, the major problem using, for example, TZD (thiazolidinedionzes) linking to the PPAR*γ* pathways still the numerous adverse effects of this kind of treatment (e.g., weight gain, anemia, leukopenia, etc.). These facts and the potential placental impacts raised also the question of the use of these medical drugs to treat the gestational diabetes. Perhaps, at the level of clinician actual knowledge, PPAR*γ* and its ligands could be used in a first time, only as good early marker candidates for the diagnosis of pregnancy pathologies like, for example, preeclampsia.

## 4. CONCLUSION

Since the discovery of the PPARs, there has been a marked increase in available data on their involvement in mammalian development. Concerning the placenta, all PPARs, but particularly PPAR*γ*, are essential for multiple physiological functions of the trophoblastic and amniotic parts, leading to major involvement of PPARs in the pathophysiology of gestational diseases. However, special care must be taken when this particular PPAR signaling cascade is involved, because part of the regulation may involve PPAR ligand signalling (by the natural 15dPGJ2 ligand or the troglitazone synthetic ligand) but may be transduced by independent nuclear receptor pathways (as, e.g., by antagonizing effects on NF-*κ*B pathway for 15dPGJ2 and by acting as an antioxidant for troglitazone). This last point introduces a new level of complexity in PPAR biology. It does not close preclusion of the eventual use of PPARs for therapeutic treatment during pregnancy, but future medical applications seem still to be a long way off. We can reasonably expect to see some obstetrical use of PPARs in diagnosis (detection of PPARs mutations in intrauterine growth retardation, predisposition of preeclampsia) and therapeutics (tocolysis or treatment of chorioamniotis).

## Figures and Tables

**Table 1:** Percentage of nucleotide and amino acid identity between the human, mouse, rat, and bovine PPAR sequences. No PPAR*γ*3 alignment was carried out owing to lack of data on different species. The different sequences came from Ensembl and were aligned with Genomatix software.

		cDNA homology (%)			Protein homology (%)		
		Mouse	Rat	Bovine	Mouse	Rat	Bovine
Human relative identity percent	PPAR*α*	44	64	72	92	92	94
	PPAR*β*	60	69	75	92	91	95
	PPAR*γ*1	79	84	78	98	97	97
	PPAR*γ*2	86	86	88	96	95	95

**Table 2:** Summary of PPAR expression patterns.

(a) Global expression pattern			
Gene	Species	Expression localization	References

PPAR*α*	Rodents	Cardiomyocytes	[6, 8]
		Hepatocytes	
		Heart	[6, 8]
		Kidney	
		Large intestine	
	Human	Leydig and seminiferous tubule cells	[12]
		Liver	[6, 8]
		Skeletal muscle	
		Uterus	[12]
		Ovary (Theca and stroma cells)	

PPAR*β*	Rodents	Ubiquitous	[6, 8]
	Human	Ubiquitous	[6, 8]

PPAR*γ*	Rodents	Brown and white adipose tissue	[6, 8]
		Lymphoid organs	
		Retina	
		Skeletal muscle	
		Uterus	[12]
		Granulosa cells, corpus luteum	
	Human	Colon	[6, 8]
		Kidney	
		Liver	
		Skeltal muscle	
		Vascular endothelium	
		White adipose tissue	
		Sertoli cells	[12]
		Uterus	
		Granulosa cells	

(B) Placental expression pattern			

PPAR*α*, *β*, *γ*	Rodents	Term placenta	[4, 9]
	Human	Villous trophoblastic cells and syncytiotrophoblasts	[4, 9, 10]
		Amnion, chorion, and amnion derived-WISH cell line	
